# Individualized anemia management in a dialysis facility – long-term utility as a single-center quality improvement experience 

**DOI:** 10.5414/CN109499

**Published:** 2018-07-27

**Authors:** Adam E. Gaweda, Alfred A. Jacobs, George R. Aronoff, Michael E. Brier

**Affiliations:** 1 Department of Medicine, University of Louisville, Louisville KY, and; 2 DaVita Kidney Care, Denver CO, USA

**Keywords:** erythropoietin, model predictive control, precision medicine, pharmacokinetics, pharmacodynamics

## Abstract

Background: Standard protocol-based approaches to erythropoiesis stimulating agent (ESA) dosing in anemia management of end-stage renal disease (ESRD) fail to address the inter-individual variability in patient’s response to ESA. We conducted a single-center quality improvement project to investigate the long-term performance of a computer-designed dosing system. Materials and methods: The study was a retrospective case-control study with long-term follow-up. All hemodialysis patients who received treatment at University Kidney Center (Louisville, KY, USA) between September 1, 2009, and March 31, 2017, were included. We implemented an individualized ESA dosing algorithm into an electronic health records database software to provide patient-specific ESA dose recommendations to anemia managers at monthly intervals. The primary outcome was the percentage of hemoglobin (Hb) concentrations between 10 and 12 g/dL during the case-control study and 9 and 11 g/dL during follow-up. Secondary outcomes were intra- and inter-individual Hb variability. For the case-control study, we compared outcomes over 12 months before and after implementation of the algorithm. Subjects served as their own controls. We used the last Hb concentration of the month and ESA dose per week. Long-term follow-up examined trends in proportion within the target range, Hb, and ESA dose. Results: Individualized ESA dosing in 56 subjects was associated with a moderate (6.6%) increase of mean Hb maintenance within target over the 12-month observation period (62.7% before vs. 69.3% after, p = 0.063). Intra-individual mean Hb variability decreased (1.1 g/dL before vs. 0.8 g/dL after, p < 0.001), so did inter-individual mean Hb variability (1.2 g/dL before vs. 1.0 g/dL after, p = 0.010). Long-term follow-up in 233 subjects for 42 months demonstrated stability of the achieved Hb despite an increasing ESA resistance in the patient population. Conclusion: Implementation of the individualized ESA dosing algorithm facilitates improvement in Hb maintenance within target, decreases Hb variability and reduces the dose of ESA required to achieve Hb target.

## Introduction 

Anemia is a frequent comorbidity in dialysis patients and is often associated with cardiovascular complications as well as decreased quality of life [1, 2]. Erythropoiesis stimulating agents (ESAs), iron supplementation, and blood transfusions are the three main forms of anemia treatment. Optimal dosing of ESA and iron to achieve a therapeutic effect without exposing the patient to risk of short- and long-term adverse events is challenging due to large inter-individual variability in response to these agents [3, 4, 5, 6, 7]. A large ESA dose and the inability to achieve target hemoglobin (Hb) concentration may be associated with increased risk of cardiovascular adverse events [1, 2, 8]. Current FDA guidelines stipulate dose individualization to minimize patient exposure to ESA while at the same time reducing the risk of transfusions [9]. Concurrently, the Quality Improvement Program metrics instituted by Centers for Medicare and Medicaid Services (CMS) stipulate maintaining Hb levels not greater than 12 g/dL in patients receiving ESA. 

Since the early days of ESA use, dialysis organizations have been striving to implement the evolving guidelines in their own anemia management protocols (AMP). A typical AMP would contain dose adjustment rules for different Hb levels, but would not explicitly take into account any measures of individual patient’s response to the drug. Different interpretation of guidelines by the providers resulted in a large variety of AMPs with different levels of efficacy. Thamer et al. [10] examined the ESA dose by dialysis provider for 2004 and saw average ESA doses ranging from 26 to 53 kU/week for hematocrit between 30 and 33%. These results exemplify the potential for dosing variability using the population approach, as well as heterogeneity in interpretation of the anemia management guidelines. 

To develop a systematic approach to individualized ESA dosing which addresses the FDA recommendations [11], we investigated the use of mathematical modeling and feedback control methods [12]. Compared to personalized medicine approaches that determine an individual’s response to treatment using patient-specific covariates, such as demographic data or proteomic and genomic biomarkers [13, 14], our approach estimates the ESA-Hb dose-response model from longitudinal ESA dose and Hb concentration data [15, 16]. The model we used was a modification of the Uehlinger model [17] and was based on parametric representation of red blood cell (RBC) production rate and RBC lifespan. Based on the model, we designed a model predictive control (MPC) algorithm to guide ESA dose adjustments over time in order to achieve a desired target Hb response in an individual patient. Through in silico simulations, we established that the algorithm was robust to short-term random Hb variability, for example due to inter-dialytic weight gain and measurement error, as well as acute adverse events, such as hemorrhage. In absence of recorded dose-response data, such as for an incident dialysis patient, the algorithm would incrementally titrate the ESA dose to achieve the dose necessary for a given Hb target as required by FDA guidelines. We tested an early version of this algorithm in two clinical studies [18, 19] and implemented it as a computerized clinical decision support system “Smart Anemia Manager” (SAM) and validated it through a randomized controlled trial (SAM RCT) [20]. We showed that individualized ESA management significantly improved Hb maintenance within target range in a select subpopulation of patients adherent to dialysis treatment and without prior diagnosis of ESA resistance. In the present study, we examine anemia parameters after 1 year of using SAM as part of a standard clinical care at our facility in an unselected patient population including subjects with different levels of response to ESA. 

## Materials and methods 

Following successful completion of the clinical trial, we introduced SAM for all in-center hemodialysis patients at our facility. The software communicates with electronic health record (EHR) database and downloads Hb and ESA dose data per patient for up to 3 months from a date specified by the user. It first performs data smoothing to calculate Hb, Hb rate of change, and average weekly ESA dose received. SAM uses this information to estimate individual patient’s dose-response profile and to compute a new ESA dose recommendation. 

**Figure 1.**
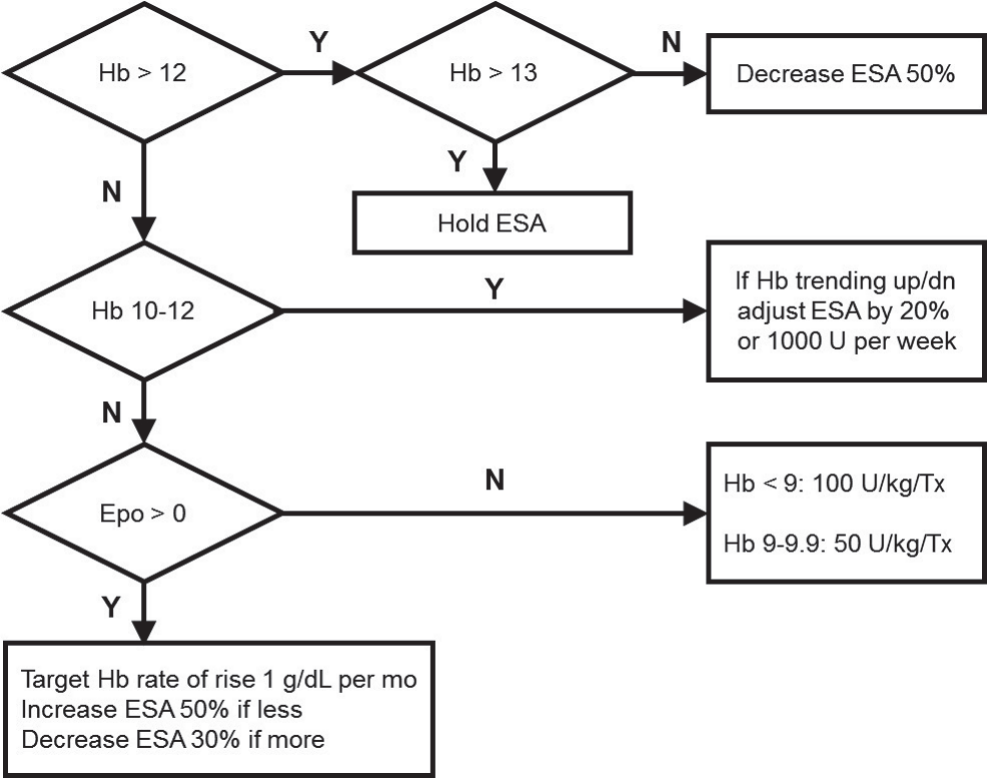
Anemia management protocol used in the control period of the study.

Consistent with the existing standard of care, Hb concentrations were measured and a qualified nurse practitioner anemia manager made ESA dose adjustments at the beginning of each month. The ESA used at the facility was epoetin alfa given by intravenous injection 1 – 3 times per week. Maximum allowed dose was 90,000 IU/week. Based on the SAM RCT findings, we introduced a minimum dose increment of 200 IU from 1,000 through 4,000 IU and a minimum dose increment of 1,000 IU above 4,000 IU. The minimum dose increment prior to introduction of SAM was 1,000 IU across the whole range. The standard of care AMP used prior to SAM RCT is shown in Figure 1. To minimize the changes to the existing clinical workflow, we did not record the deviations from SAM-recommended doses, as such deviations were not recorded prior to introduction of SAM. As an emergency backup, in case of EHR failure, we generated a simplified version of SAM algorithm in the form of dose adjustment look-up tables that were maintained by the nurse managers in a binder. 

Intravenous iron sucrose was administered per facility-specific iron protocol derived from the National Kidney Foundation Kidney Disease Outcomes Quality Initiative guidelines. Iron sucrose was administered to maintain ferritin levels greater than 300 ng/mL but not exceeding 800 ng/mL and TSAT greater than 30%. The maximum iron dose was 100 mg 3 times per week. 

We performed two separate analyses of anemia management using SAM a) a retrospective longitudinal case-control study of ESRD patients receiving hemodialysis treatments at University of Louisville Kidney Center; b) long-term follow-up of anemia management. The research protocol conformed to the Declaration of Helsinki and was approved by the University of Louisville Institutional Review Board. All subject data were de-identified. Eligible participants were prevalent hemodialysis patients who received in-center treatment 3 times a week between September 1, 2009, and March 30, 2017. The study time periods were as follows:

- Protocol control from September 1, 2009, through August 31, 2010
- Exclusion period during the SAM RCT [20] September 1, 2010, through March 31, 2012
- Post-study washout period from April 1, 2012, through July 13, 2012
- SAM dosing from August 1, 2012, through July 31, 2013
- Long-term follow-up from October 1, 2013 (the first full quarter following the SAM trial), through March 31, 2017

To be included in the case-control analysis, patients had to have received ESA in both study periods. The Hb target was consistent at 10 – 12 mg/dL during the entire study period. All subjects who received an ESA within 3 months were included in the long-term follow-up. 

The primary outcome was the proportion of Hb concentrations between 10 and 12 g/dL over each of the study periods (12 months) calculated using monthly Hb measurements in the case-control study and the proportion of Hb concentrations between 9 and 11 g/dL in the follow up study where the target range was adjusted following national recommendation. If a subject had repeated Hb measurements within a month, the last measurement was used for analysis, consistent with the CMS reporting guidelines. Secondary outcomes were the proportions of Hb below or above the stated target range. Additional outcomes for the case-control study included mean Hb achieved, intra-individual Hb variability, inter-individual Hb variability, weekly ESA dose, weekly iron dose, dialysis adequacy (Kt/V), serum albumin, serum ferritin, transferrin saturation (TSat), serum calcium, serum phosphorus, parathryoid hormone (PTH), mean corpuscular hemoglobin (MCH), mean corpuscular hemoglobin concentration (MCHC), mean corpuscular volume (MCV), and the percentage of completed dialysis treatments. 

### Case-control analysis 

Each subject served as their own control resulting in pairwise comparison of outcomes. Statistical comparison was performed using paired t-test for normally distributed variables and Wilcoxon signed rank test for non-normally distributed variables. Normally distributed variables were reported as mean and standard deviations, whereas non-normally distributed variables were reported as median and range (min, max). All calculations were performed in SPSS Statistics Ver. 24 (IBM). To investigate the association between ESA dose and anemia parameters we performed multiple linear regression analysis. We used log10-transformed ESA dose as the dependent variable and study period, mean Hb, intra- and inter-individual Hb variability, Kt/V, albumin, calcium, phosphorus, TSat, log10-transformed ferritin, log10-transformed PTH, MCH, MCHC, MCV, log10-transformed cumulative iron dose, and the percentage of completed treatments as independent covariates. 

### Follow-up study 

All subjects dialyzed in this single facility were included in this retrospective analysis over 42 months. The primary outcome was the proportion of Hb values within the target range of 9 – 11 g/dL. Secondary outcomes were the average ESA dose received and compliance with the dialysis prescription defined as receiving > 90% of the prescribed time and the receiving the prescribed ESA dose. Hb values were included as the last measured Hb during the month and the subject had received an ESA within 3 months of the current measurement. Regression analysis was preformed on Hb concentration and ESA dose over time. 

## Results 

### Case-control study 

**Figure 2.**
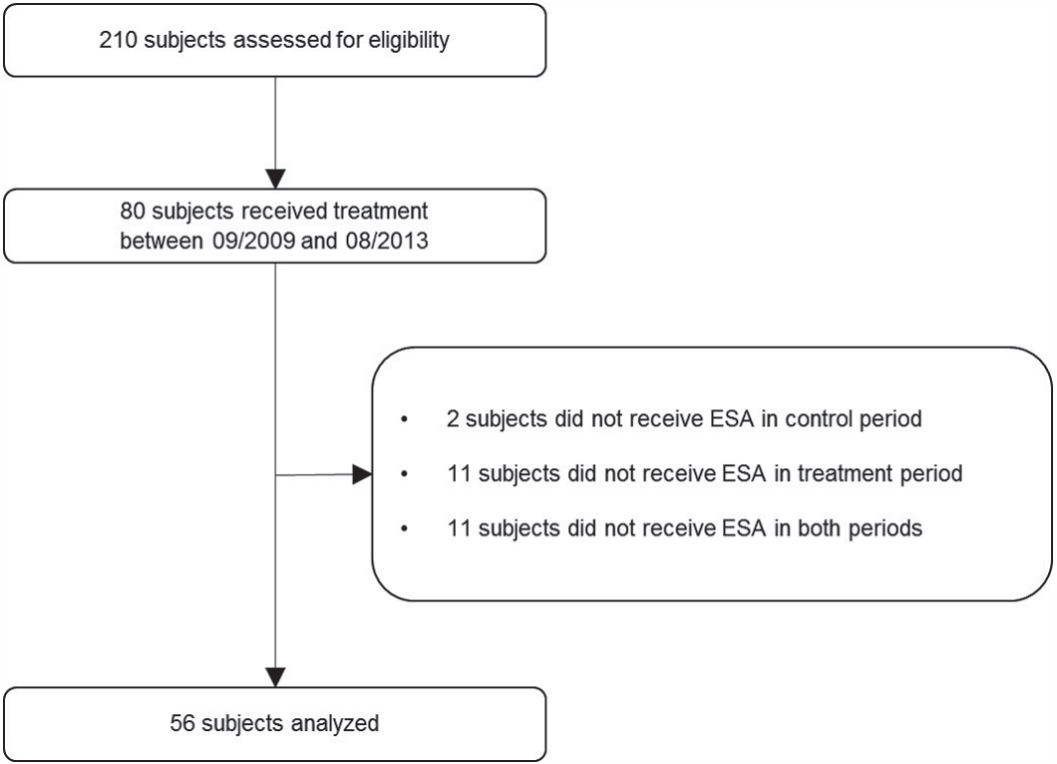
Consolidated standards of reporting trials diagram of participant flow through the study.

Out of the 210 patients screened, 80 subjects received treatment between September 1, 2009, and July 31, 2013. Out of the 80 subjects, 2 did not receive ESA in the protocol period, 11 did not receive ESA in the SAM period, and 11 did not receive ESA within any of the study periods, resulting in a final sample of 56 subjects (Figure 2). Demographic and clinical data of the study population are shown in Table 1. 

**Figure 3.**
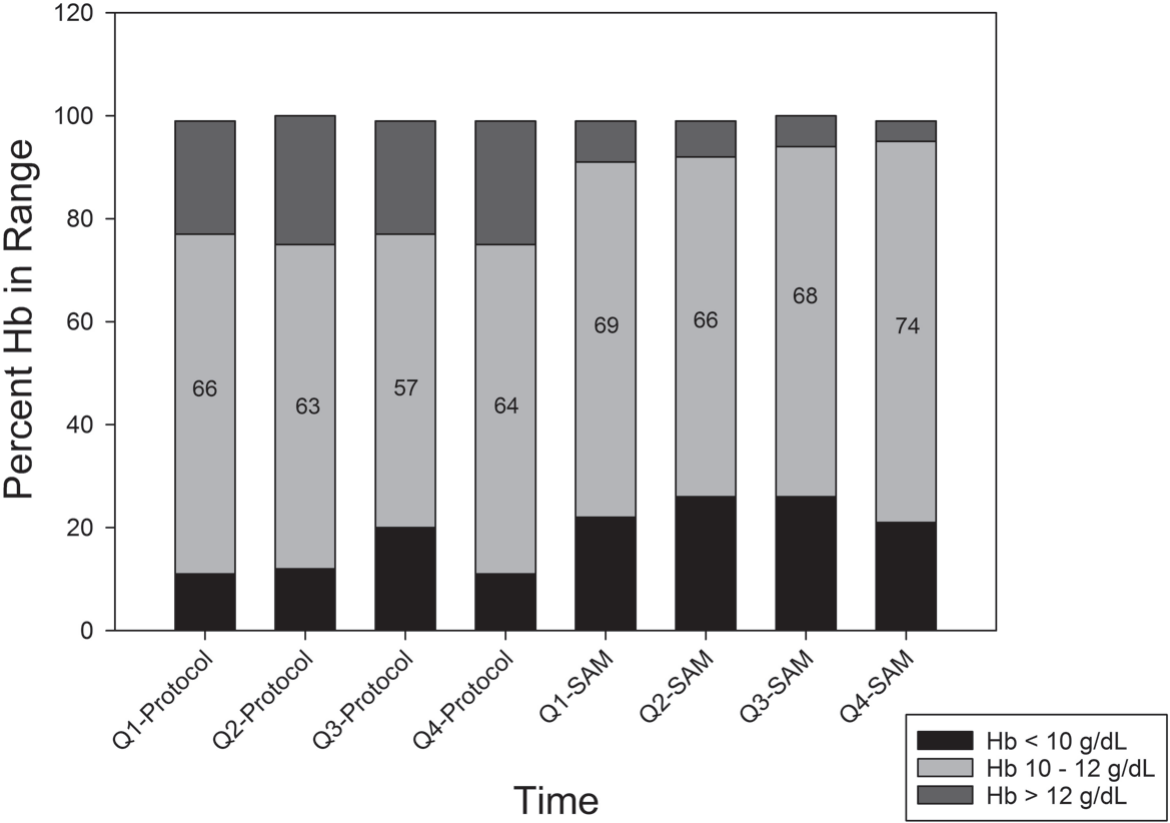
Hemoglobin distribution per quarter during the case-control study: below/within/above target percentage bar plot. Numbers within the box plot show percentage within the target range of 10 – 12 mg/dL.

Statistical comparison of the anemia management outcomes between the study periods is shown in Table 2. SAM-guided ESA dosing resulted nearly 70% of Hb measurements within the 10 – 12 g/dL range, compared to ~ 63% when dosing per AMP (p = 0.063). Compared to AMP-based approach, SAM-guided dosing increased the number of Hb measurements less than 10 g/dL by ~ 10% (p = 0.006) and decreased the number of Hb measurements greater than 12 g/dL by ~ 17% (p < 0.001). The number of Hb measurements less than 9 g/dL remained comparable between the two methods (p = 0.184). Mean achieved Hb was 10.5 g/dL for SAM-guided dosing, compared to 11.2 g/dL for AMP-based dosing (p < 0.001). Figure 3 shows a bar plot of quarterly Hb percentages below, within, and above the 10 – 12 g/dL range during the study. 

Dialysis adequacy (Kt/V), albumin, TSat, calcium, phosphorus, MCH, MCHC, and MCV remained comparable during both study periods. However, ferritin and parathyroid hormone were greater in the treatment period. The percentage of completed treatments (adherence) was not statistically different between the study periods. Linear regression analysis revealed that the percentage of completed treatments was a predictor of mean achieved Hb and ESA dose administered. Each missed dialysis treatment was associated with a decrease in mean Hb by 0.01 g/dL (p < 0.001) in the protocol period and 0.02 g/dL (p < 0.001) in the SAM period. Furthermore, each missed treatment was associated with an increase in the weekly ESA dose received of 10 IU (p = 0.011) in the protocol period and 4.5 IU (p = 0.109) in the SAM period. 

Comparison of the ESA dose between the two study periods revealed over a 2-fold decrease in utilization under SAM-guided dosing compared to the AMP-based approach. The median ESA dose decreased from 7,800 to 3,000 U/week (p < 0.001). The corresponding decrease in mean ESA dose was 12,650 – 6,300 U/week. A similar decrease was also observed for intravenous iron dose. The median iron dose decreased from 43 to 16 mg/week (p < 0.001). The corresponding decrease in mean iron dose was 51 – 25 mg/week. 

We found that mean Hb, intra-individual Hb variability, phosphorus, and TSat were statistically associated with the ESA dose. Using estimated model coefficients, we evaluated the relative impact of the change in these covariates on the dose reduction. The 0.7 g/dL decrease in mean Hb contributed 34% (p < 0.001), the 0.3 g/dL decrease in Hb variability contributed 16% (p = 0.001), the 0.2 mg/dL decrease in phosphorus contributed 3.5% (p < 0.001), the 1.9% increase in TSat contributed 3.1% (p = 0.05), and 43.3% was unexplained toward the observed ESA dose reduction, respectively. 

There was no difference in hospital stays between the two study periods. There were a total of 89 hospitalizations in the protocol period vs. 74 in the SAM period (p = 0.608). Subjects spent a total of 498 days in hospital in the protocol period vs. 420 in the SAM period (p = 0.812). 

### Follow-up study 

**Figure 4.**
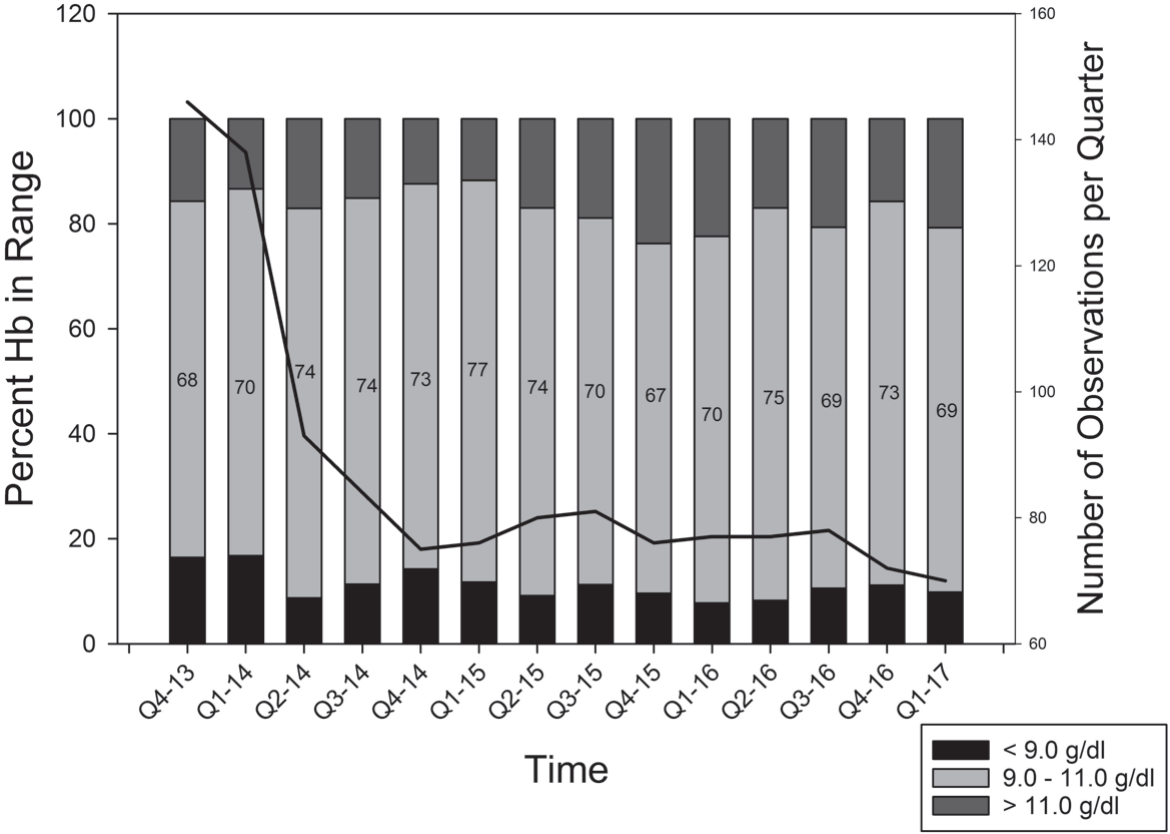
Hemoglobin distribution per quarter during long-term follow-up: below/within/above target percentage bar plot. Numbers within the box plot show percentage within the target range of 9 – 11 mg/dL. Solid line displays the number of patients receiving an ESA within 3 months.

**Figure 5.**
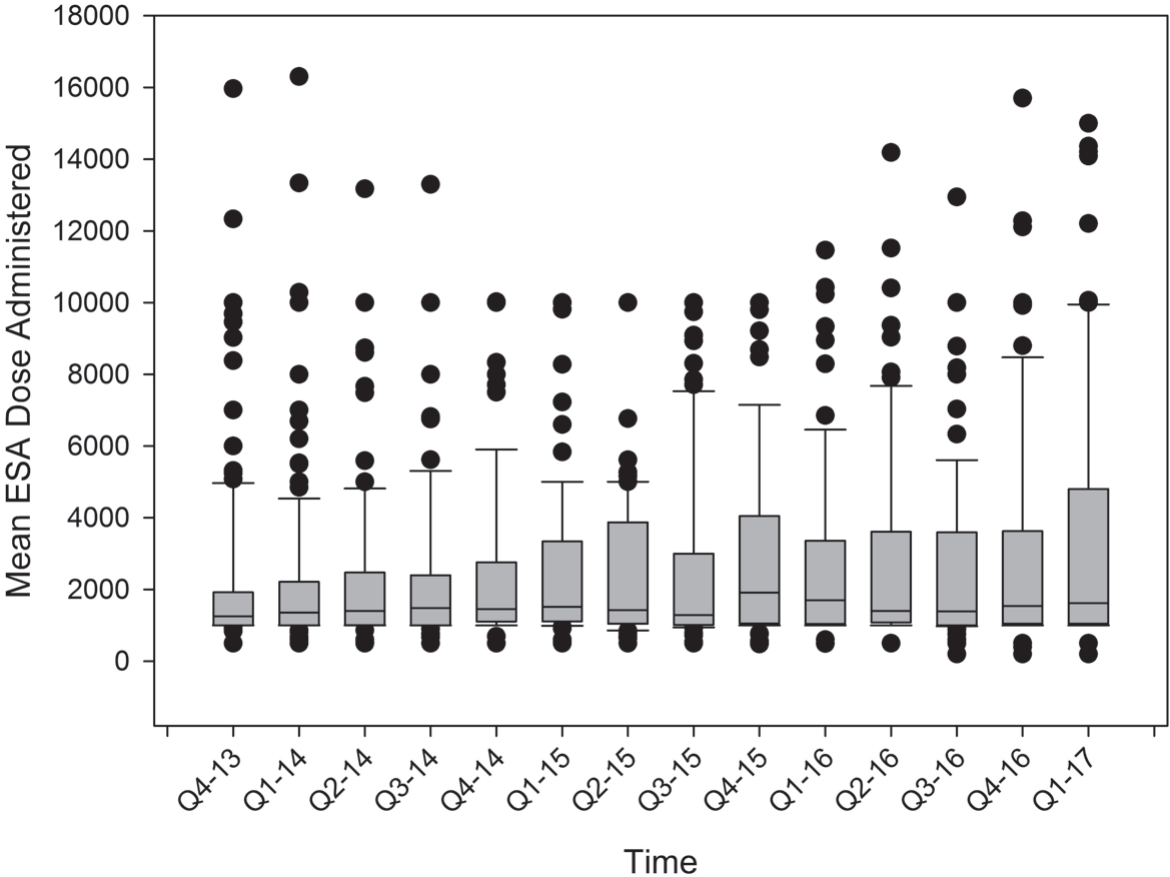
Box plot of the mean ESA dose received per quarter during the long-term follow-up.

Demographic information on the 233 subjects included in the follow-up period are shown in Table 1. Achieved Hb concentrations during the follow-up period are displayed in Figure 4 as the percent below, within, and above the target range. Long-term follow-up in an unselected population of subjects resulted in a mean monthly Hb achieved of 10.04 g/dL ranging from 9.70 to 10.39 g/dL with a resulting standard deviation of 1.11 g/dL ranging from 0.78 to 1.35 g/dL. During the 42 months of follow-up, the mean Hb increased at a rate of 0.084 g/dL a year (p = 0.002). Likewise, ESA dose increased during the follow-up period from a mean of 2,180 units in the 4^th^ quarter of 2013 to 3,478 units in the first quarter of 2017. The standard deviation ranged from 2,135 to 3,787 units. The data are displayed in Figure 5. Long-term follow-up resulted in a mean administered ESA dose of 3,051 units ranging from 2,315 to 4,176 units with a resulting mean standard deviation of 2,777 units ranging from 2,143 to 4,275 units. During the 42 months of follow-up, the mean ESA dose increased at a rate of 389 units a year (p < 0.001). 

The percent of incomplete (< 90% of the prescribed treatment time) or missed dialysis treatments remained constant at 15.7% (13.0 – 18.3%) during the follow-up period. The percent of scheduled ESA doses not administered also remained constant at 11.4% (9.1 – 13.6%). 

## Discussion 

In January 2010, the FDA called for clinical trials to establish optimal ESA dosing algorithms including computer-directed algorithms [11]. We developed a CDSS software (SAM) which implements an algorithm based on the principles of mathematical modeling and control theory to derive precise ESA dose recommendations for individual patients from standard clinical data stored in EHR. Previously, we demonstrated through a randomized controlled trial (SAM RCT) that SAM-guided individualized ESA dosing improved Hb control compared to an AMP-based approach [20]. Because SAM RCT focused on a select group of patients adherent to the dialysis treatment regimen and responsive to ESA, the question remained how well the SAM software would perform when implemented as part of standard care in all patients, including patients resistant to ESA and patients with low treatment adherence. To answer this question, we performed a longitudinal retrospective case-control analysis of the commonly used anemia markers following the implementation of the SAM software as a standard ESA dosing approach in all of our in-center hemodialysis patients receiving ESA. Consistent with the previously reported results [20], we observed an increase in the proportion of Hb measurements within the target range 10 – 12 g/dL (the recommended target range at the time) compared to a standard protocol-based approach previously used at our facility. We note that SAM-guided ESA dosing significantly reduced the number of “high” (> 12 g/dL) Hb concentrations. Although this reduction came with an increase in Hb < 10 g/dL, the number of “low” (< 9 g/dL) Hb concentrations remained comparable. 

As expected, the observed effect of individualized ESA dosing on Hb variability in the present study did not result in the same level of decrease as the one reported in the SAM RCT [20]. Our analysis shows that missed dialysis treatments may have an effect on both, the mean Hb concentration achieved and the ESA dose. We found that each missed dialysis treatment was associated with a decrease in Hb concentration and this effect was stronger (1.8 times) during the SAM period compared to the protocol period. On the other hand, each missed dialysis treatment was associated with an increase in ESA dose and was stronger (2.3 times) during the protocol period. These effects were most pronounced in patients who completed less than 80% of their treatments. We think that this observation can be partially attributed to the use of the average dose received as the basis for new dose calculation in SAM, whereas the AMP used the dose prescribed. In our opinion, optimal ESA dosing in patients who tend to miss their dialysis treatments requires further investigation. 

In SAM RCT, we did not observe an ESA dose difference effect between study groups. However, we did observe a decrease in ESA dose compared to the national average at the time [21]. In the present study, we observed a greater than 50% decrease in ESA dose, compared to the previously used AMP. The ESA dose achieved by SAM-guided dosing in the present study was consistent with that reported in SAM RCT [20]. The observed decrease in ESA dose was primarily associated with lower mean Hb achieved. However, we found that smaller intra-individual Hb variability, higher TSat, and lower phosphorus were also related to lower ESA dose. Whereas the effects of TSat [22, 23] and phosphorus [24] on ESA dose have been reported before, the association between intra-individual Hb variability and ESA dose is yet to be explored. One possible explanation for the observed relationship between intra-individual Hb variability and ESA dose could be that SAM-guided dose adjustments were in general smaller when compared to the recommended 25% dose change routinely used in an anemia management protocol. Small dose adjustments may lead to slower Hb changes and lower total ESA dose over time. This is speculation on our part and would need to be confirmed. 

Routinely monitored iron indices as well as iron dose delivered were not different in SAM RCT [20]. In the present study, we observed no difference in TSat, but we did observe an increased ferritin concentration and a decrease in iron dose in the treatment period. The increase in ferritin may be a result of ~ 2 years of additional hemodialysis, iron administration, and disease progression [25]. Furthermore, the rise in ferritin may have also led to the 50% decrease in intravenous iron administration observed. The issue of coordinated dosing of ESA and iron is a subject of our ongoing investigation. 

Long-term use of the SAM protocol was examined from the 4^th^ quarter of 2013 until the 1^st^ quarter of 2017. During this time, two disturbances occurred in the study population, 1) the target range was changed to 9 – 11 g/dL and 2) the dialysis facility was sold and some patients moved to one of three new dialysis facilities. The SAM protocol does not require reprogramming to accommodate a change in target and the population achieved the new target within 3 months and with similar results (percent of subjects within the target range). However, the resistance to the erythropoietic effect of an ESA did increase as demonstrated by the progressive rise in ESA dose. This increase cannot be explained by adherence to dialysis therapy or missing ESA doses as these do not change during follow-up but may be due to the “aging” of the patient population with dialysis vintage increasing from 4.6 to 7.3 years. 

SAM is not the only tool that uses mathematical models to guide ESA dosing. Lines et al. [26] developed a predictive dose-response model to derive ESA dose adjustment rules. A prospective evaluation of anemia management parameters supported by this model revealed improvement of Hb maintenance within the target range (66% in 10.5 – 12.5 g/dL). A predictive algorithm for ESA dosing was presented by McCarthy et al. [27]. In this approach, an individualized physiologic model of erythropoiesis identified from treatment data in EHR (MCAMS) guided the dose selection of darbopoetin. Retrospective analysis of MCAMS-based anemia management showed an improvement in Hb maintenance within the target range (83% in 10 – 12.9 g/dL) and a 40% decrease in darbepoetin use. Barbieri et al. [28] showed that the implementation of a predictive model based on artificial neural network approach at multiple dialysis facilities resulted in improvement of Hb proportion within target range and ESA dose savings in hemodialysis patients. Our results further strengthen this trend. 

Compared to the above approaches, where predictive models are used in an open-loop fashion, our methodology combines predictive modeling with closed-loop control, in our case MPC. One important benefit of using closed-loop control is the ability to account for Hb concentration errors due to factors other than ESA, for example inter-dialytic fluid changes. Current clinical standards of care for anemia management do not explicitly account for the effect of inter-dialytic fluid change on Hb concentration, which may lead to inadvertent ESA dose adjustments in response to increased or decreased Hb dilution. In our approach, we used the robustness property of closed-loop control to address this challenge by modeling inter-dialytic fluid gains as parametric uncertainty affecting the Hb concentration. 

We think that the precision of our algorithm will be improved once explicit measures of inter-dialytic fluid change are added to the model. 

Our study has several limitations. First, it is an observational study, therefore all the findings have an associative character. Because of the longitudinal nature of the study, we cannot fully exclude the effect of disease progression on the study outcomes. The time effect should be kept in consideration when interpreting the results. Transfusion data were not routinely reported before 2011. Since all transfusions occurred outside our dialysis unit, this made a retrospective review unreliable. Another limitation of the present study is the absence of data on the nurse adherence to dose recommendations. Dose deviations are not collected per standard clinical procedures and were not included in the study design in order to streamline the implementation process. 

## Conclusion 

Individualized ESA dosing is needed to help achieve anemia management outcomes consistent with national guidelines. We confirm the results of our previously published randomized controlled study and show that individualized ESA dosing decreases Hb variability and minimizes patient exposure to the drug. Further validation in a multi-center setting will be required to further confirm the utility of this approach. 

## Declaration 

Human Studies Authorization: The collection of data was approved by the University of Louisville Institutional Review Board 14.01.13 Retrospective Analysis of Anemia Management Patterns and Outcomes 

## Funding 

Research reported in this publication was supported by the National Institute of Diabetes And Digestive And Kidney Diseases of the National Institutes of Health under Award Number R01DK093832 and R01DK091584. The content is solely the responsibility of the authors and does not necessarily represent the official views of the National Institutes of Health. 

## Conflict of interest 

The authors are the inventors of the SAM software and have a patent application for the technology. The SAM software is licensed by Dosis Inc. and the authors (Gaweda, Aronoff, Brier) are on the Scientific Advisory Board. 

**Table 1.** Patient demographics.

Case-control study	
Age (years),mean [range]	59 [26,90]
Sex (m/f)	28/28
Race	
Caucasian	11
African-American	45
Dialysis vintage (years) mean [range]	4 [0,20]
Long-term follow-up study	
Age (years) mean [range]	
2013	57.9 [12,94]
2014	57.6 [12,95]
2015	55.6 [9,96]
2016	54.6 [10,97]
2017	56.0 [17,98]
Sex (m/f)	138/95
Race	
Caucasian	53
African-American	176
Other	4
Dialysis vintage (years) mean [range]	
2013	4.6 [0,27]
2014	5.2 [0,28]
2015	6.2 [0,35]
2016	6.7 [0,27]
2017	7.3 [0,28]

**Table 2.** Statistical comparison of study outcomes.

Outcome	Period		p-value
	Protocol	SAM	
Percentage Hb 10 – 12 g/dL*	62.7	69.3	0.063^1^
Percentage Hb < 9 g/dL	4.1	7.6	0.184^1^
Percentage Hb < 10 g/dL	13.9	24.2	0.006^1^
Percentage Hb > 12 g/dL	23.4	6.5	< 0.001^1^
Hb (g/dL)	11.2 ± 0.5	10.5 ± 0.6	< 0.001^2^
Intra-individual Hb variability (g/dL)	1.1 [0.4, 2.4]	0.8 [0.2, 1.8]	< 0.001^1^
Inter-individual Hb variability (g/dL)	1.2 [1.0, 1.5]	1.0 [0.9, 1.2]	0.010^1^
Kt/V	1.6 ± 0.2	1.5 ± 0.2	0.277^2^
Serum albumin (g/dL)	3.9 ± 0.3	3.9 ± 0.2	0.305^2^
Transferrin saturation (%)	30.8 ± 6.0	32.7 ± 8.6	0.135^2^
Serum ferritin (ng/mL)	845 [179, 1,759]	1,071 [62, 3,319]	< 0.001^1^
Serum calcium (g/dL)	8.8 ± 0.6	8.9 ± 0.5	0.198^2^
Serum phosphorus (mg/dL)	5.8 ± 1.3	5.6 ± 1.4	0.614^2^
PTH (pg/mL)	398 [30, 2107]	501 [52, 4,106]	0.012^1^
MCH (pg)	30.2 ± 2.6	30.6 ± 2.6	0.397^2^
MCHC (g/dL)	32.2 ± 1.1	31.9 ± 0.9	0.237^2^
MCV (fL)	93.7 ± 6.8	95.7 ± 7.2	0.135^2^
Weekly ESA dose (IU)	7,800 [1,000, 58,000]	3,000 [1,000, 35,000]	< 0.001^1^
Weekly iron dose (mg)	43 [0, 211]	16 [0,102]	< 0.001^1^
Percentage completed treatments	90	91	0.582^1^

Normally distributed variables reported as mean ± standard deviation. Non-normally distributed variables reported as median [min, max]. Hb = hemoglobin; PTH = parathyroid hormone; MCH = mean corpuscular hemoglobin; MCHC = mean corpuscular hemoglobin concentration; MCV = mean corpuscular volume. *Primary outcome; ^1^Wilcoxon signed rank test; ^2^Paired sample t-test.
